# Complete genome sequence of *Mycobacterium sp.* strain (Spyr1) and reclassification to *Mycobacterium gilvum* Spyr1

**DOI:** 10.4056/sigs.2265047

**Published:** 2011-10-01

**Authors:** Aristeidis Kallimanis, Eugenia Karabika, Kostantinos Mavromatis, Alla Lapidus, Kurt M. LaButti, Konstantinos Liolios, Natalia Ivanova, Lynne Goodwin, Tanja Woyke, Athanasios D. Velentzas, Angelos Perisynakis, Christos C. Ouzounis, Nikos C. Kyrpides, Anna I. Koukkou, Constantin Drainas

**Affiliations:** 1 Sector of Organic Chemistry and Biochemistry, University of Ioannina, 45110 Ioannina, Greece; 2 DOE Joint Genome Institute, Walnut Creek, California, USA; 3 Los Alamos National Laboratory, Bioscience Division, Los Alamos, New Mexico, USA; 4 Department of Cell Biology and Biophysics, Faculty of Biology, University of Athens, 15701, Athens, Greece; 5 Centre for Bioinformatics, Department of Informatics, School of Natural & Mathematical Sciences, King's College London (KCL), London WC2R 2LS, UK; § Present address: Computational Genomics Unit, Institute of Agrobiotechnology, Center for Research & Technology Hellas (CERTH), GR-57001 Thessaloniki, Greece & Donnelly Centre for Cellular & Biomolecular Research, University of Toronto, 160 College Street, Toronto, Ontario M5S 3E1, Canada

**Keywords:** *Mycobacterium gilvum*, PAH biodegradation, pyrene degradation

## Abstract

*Mycobacterium* sp.Spyr1 is a newly isolated strain that occurs in a creosote contaminated site in Greece. It was isolated by an enrichment method using pyrene as sole carbon and energy source and is capable of degrading a wide range of PAH substrates including pyrene, fluoranthene, fluorene, anthracene and acenapthene. Here we describe the genomic features of this organism, together with the complete sequence and annotation. The genome consists of a 5,547,747 bp chromosome and two plasmids, a larger and a smaller one with sizes of 211,864 and 23,681 bp, respectively. In total, 5,588 genes were predicted and annotated.

## Introduction

Strain Spyr1 (=LMG 24558, =DSM 45189) is a new strain which based on its morphological and genomic features, belongs to the genus *Mycobacterium* [1]. It was isolated from Perivleptos, a creosote polluted site in Epirus, Greece (12 Km North of the city of Ioannina), where a wood preserving industry was operating for over 30 years. Strain Spyr1 is of particular interest because it is able to utilize a wide range of PAH substrates as sole sources of carbon and energy, including pyrene, fluoranthene, fluorene, anthracene and acenapthene. Microbial degradation is one of the major routes by which Polycyclic Aromatic Hydrocarbons (PAHs) can be removed from the environment. Strain Spyr1 metabolizes pyrene to 1-Hydroxy-2-naphthoic acid which subsequently is degraded via *o*-phthalic acid, a pathway also proposed for other *Mycobacterium* strains [1] exhibiting desirable PAH degradation properties as follows. Complete degradation of pyrene at concentrations 80 mg/L occurred within eight days of incubation in the dark [1]. The extrapolated degradation rate for the growth-phase can be averaged to 10 gml^-1^day^-1^, a value similar to that reported for other *Mycobacterium* species [2,3]. Addition of vitamins or trace amounts of yeast extract were not required for the growth of Spyr1 on any PAH, unlike other *Mycobacterium* spp. [4]. Use of free or entrapped cells of strain Spyr1 resulted in total removal of PAH from spiked soil samples [1]. Here a summary classification and a set of features for strain Spyr1, along with the description of the complete genome sequence and annotation are presented.

## Classification and Features

**Figure 1:**
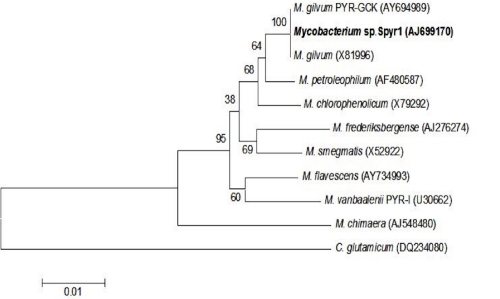
Phylogenetic location of strain Spyr1 among other *Mycobacterium* species. *Corynebacterium glutamicum* was used as the outgroup. The scale bar indicates the number of substitutions per nucleotide position (Number of bootstrap analysis: 1000).

The phylogenetic tree of strain Spyr1 according to 16S rDNA sequences is depicted in Figure 1.

**Figure 2:**
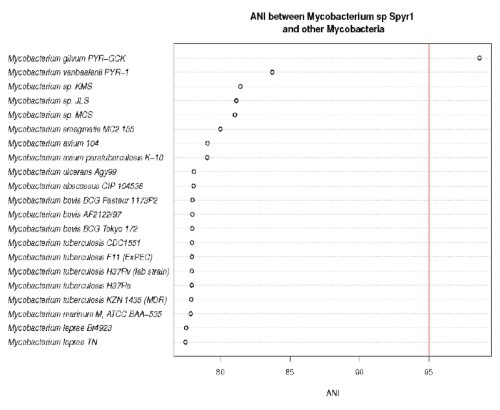
ANI values between *Mycobacterium* sp. Spyr1 and other Mycobacteria. The red line is drawn at ANI 95 a suggested threshold for species.

The sequence identity of the 16S rRNA genes of strain Spyr1 to those from the two *M. gilvum* strains is 99%, while the average nucleotide identity (ANI) [5] between strain Spyr1 and *M. gilvum* PYR-GCK is 98.5. This information indicates that Spyr1 is a strain of *M gilvum*. Accordingly, we propose the renaming of the Spy1 strain to *M. gilvum* Spyr1. The ANI values between strain Spyr1 and other sequenced Mycobacteria are depicted in Figure 2.

**Figure 3:**
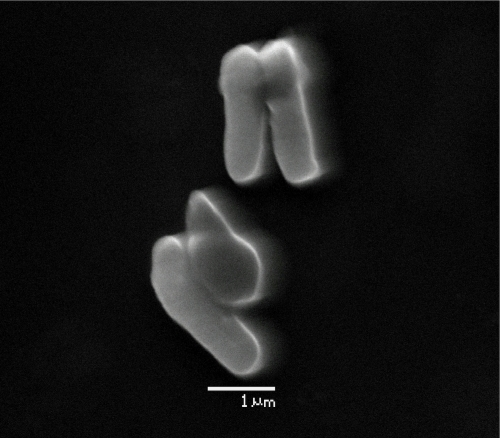
Scanning electron micrograph of *Mycobacterium gilvum* strain Spyr1.

Strain Spyr1 is an aerobic, non-motile rod, with a cell size of approximately 1.5-2.0 × 3.5-5.0 μm and produces only a weakly positive result under Gram staining. (Figure 3). Colonies were slightly yellowish on Luria agar. The temperature range for growth was 4-37°C with optimum growth at 30-37°C. The pH range was 6.5-8.5 with optimal growth at pH 7.0-7.5. Strain Spyr1 was found to be sensitive to various antibiotics, the minimal inhibitory concentrations were reported as follows: chlorampenicol 10 mgL^-1^, erythromycin 10 mgL^-1^, rifampicin 10 mgL^-1^ and tetracycline 10 mgL^-1^.

Catalase and nitrate reductase tests were positive, whereas arginine dihydrolase, gelatinase, lipase, lysine and ornithine decarboxylase, oxidase, urease, citrate assimilation and H_2_S production tests were negative. No acid was produced in the presence of glucose, lactose, sucrose, arabinose, galactose, glycerol, *myo*-inositol, maltose, mannitol, raffinose, sorbitol, sucrose, trehalose and xylose (see also Table 1).

**Table 1:** Classification and general features of strain Spyr1 according to the MIGS recommendations [6]

MIGS ID	Property	Term	Evidence code
	Current classification	Domain *Bacteria*	TAS [7]
		Phylum *Actinobacteria*	TAS [8]
		Class *Actinobacteria*	TAS [9]
		Subclass *Actinobacteridae*	TAS [9,10]
		Order *Actinomycetales*	TAS [9-12]
		Suborder *Corynebacterineae*	TAS [9,10]
		Family *Mycobacteriaceae*	TAS [9-11,13]
		Genus *Mycobacterium*	TAS [11,14,15]
		Species *Mycobacterium gilvum*	TAS [11,13]
		strain Spyr1	TAS [1]
	Gram stain	Weakly positive	TAS [1]
	Cell shape	irregular rods	TAS [1]
	Motility	Non motile	TAS [1]
	Sporulation	nonsporulating	NAS
	Temperature range	mesophile	TAS [1]
	Optimum temperature	30°C	TAS [1]
	Salinity	normal	TAS [1]
MIGS-22	Oxygen requirement	aerobic	TAS [1]
	Carbon source	Pyrene, fluoranthene, phenanthrene, anthracene, glucose, yeast extract	TAS [1]
	Energy source	Pyrene, fluoranthene, phenanthrene, anthracene, glucose, yeast extract	TAS [1]
MIGS-6	Habitat	Soil	TAS [1]
MIGS-15	Biotic relationship	Free-living	NAS
MIGS-14	Pathogenicity	none	NAS
	Biosafety level	1	NAS
	Isolation	Creosote contaminated soil	TAS [1]
MIGS-4	Geographic location	Perivleptos, Epirus, Greece	TAS [1]
MIGS-5	Sample collection time	April 2000	TAS [1]
MIGS-4.1	Latitude	39.789	NAS
MIGS-4.2	Longitude	20.781	NAS
MIGS-4.3	Depth	10-20 cm	TAS [1]
MIGS-4.4	Altitude	500 m	TAS [1]

Evidence codes - TAS: Traceable Author Statement (i.e., a direct report exists in the literature); NAS: Non-traceable Author Statement (i.e., not directly observed for the living, isolated sample, but based on a generally accepted property for the species, or anecdotal evidence). These evidence codes are from of the Gene Ontology project [16].

### Chemotaxonomy

Strain Spyr1 major fatty acids are C_16:1_ (16.7%), C_16:0_ (32,9%), C_18:1_(47.5%), C_18:0_ (1.0%) and C_19:0_ cyclo(1.1%). The major phospholipids were phosphatidylethanolamine (PE), phosphatidylglycerol (PG) and diphospatidylglycerol (DPG) (80.4, 4.7 and 15.0% respectively).

## Genome sequencing information

### Genome project history

This organism was selected for sequencing on the basis of its biodegradation capabilities, i.e. metabolizes phenanthrene as a sole source of carbon and energy. The genome project is deposited in the Genome OnLine Database [17] and the complete genome sequence is deposited in GenBank. Sequencing, finishing and annotation were performed by the DOE Joint Genome Institute (JGI). A summary of the project information is shown in Table 2.

**Table 2:** Genome sequencing project information

**MIGS ID**	**Property**	**Term**
MIGS-31	Finishing quality	Finished
MIGS-28	Libraries used	Tree genomic libraries: Sanger 9 kb pMCL200, fosmids and 454 standard library
MIGS-29	Sequencing platforms	ABI3730, 454 GS FLX
MIGS-31.2	Sequencing coverage	10.26 × Sanger; 43.3 × pyrosequence
MIGS-30	Assemblers	Newbler version 1.1.02.15, Arachne
MIGS-32	Gene calling method	Prodigal 1.4, GenePRIMP
	Genbank ID	CP002385, CP002386, CP002387
	Genbank Date of Release	December 21, 2010
	GOLD ID	Gc01567
	NCBI project ID	28521
	Database: IMG	649633070
MIGS-13	Source material identifier	DSM 45189
	Project relevance	Bioremediation, PAH degradation

### Growth conditions and DNA isolation

*Mycobacterium gilvum* Spyr1, DSM 45189 was grown aerobically at 30°C on MM M9 containing 0.01% (w/v) pyrene. DNA was isolated according to the standard JGI (CA, USA) protocol for bacterial genomic DNA isolation using CTAB.

### Genome sequencing and assembly

The genome of *Mycobacterium gilvum* Spyr1 strain was sequenced using a combination of Sanger and 454 sequencing platforms. All general aspects of library construction and sequencing can be found at the JGI website [18]. Pyrosequencing reads were assembled using the Newbler assembler version 1.1.02.15 (Roche). Large Newbler contigs were broken into 6,290 overlapping fragments of 1,000 bp and entered into assembly as pseudo-reads. The sequences were assigned quality scores based on Newbler consensus q-scores with modifications to account for overlap redundancy and to adjust inflated q-scores. A hybrid 454/Sanger assembly was made using the Arachne assembler [19]. Possible mis-assemblies were corrected and gaps between contigs were closed by editing in Consed, with custom primer walks from sub-clones or PCR products. A total of 346 Sanger finishing reads were produced to close gaps, resolve repetitive regions, and raise the quality of the finished sequence. The error rate of the completed genome sequence is less than 1 in 100,000. Together, the combination of the Sanger and 454 sequencing platforms provided 53.56 x coverage of the genome. The final assembly contains 61,443 Sanger reads and 1,300,893 pyrosequencing reads.

### Genome annotation

Genes were identified using Prodigal [20] as part of the Oak Ridge National Laboratory genome annotation pipeline, followed by a round of manual curation using the JGI GenePRIMP pipeline [21]. The predicted CDSs were translated and used to search the National Center for Biotechnology Information (NCBI) nonredundant database, UniProt, TIGR-Fam, Pfam, PRIAM, KEGG, COG, and InterPro databases. Comparative analysis was performed within the Integrated Microbial Genomes (IMG) platform [22].

## Genome properties

**Figure 4:**
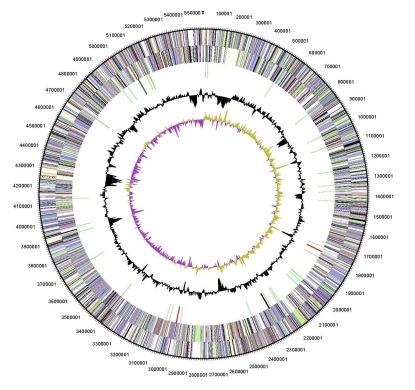
Graphical circular map of the chromosome of strain Spyr1. From outside to the center: Genes on forward strand (color by COG categories), Genes on reverse strand (color by COG categories), RNA genes (tRNAs green, rRNAs red, other RNAs black), GC content, GC skew.

**Figure 5:**
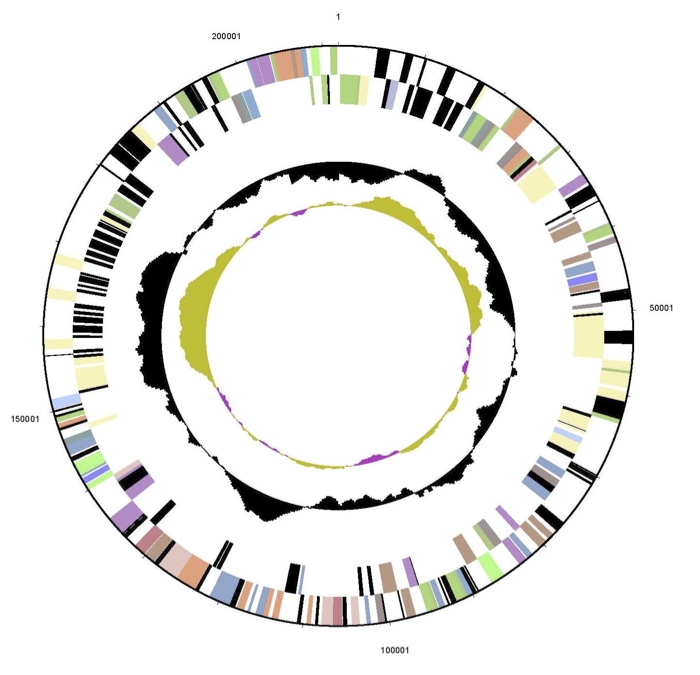
Graphical circular map of first plasmid of strain Spyr1. From outside to the center: Genes on forward strand (color by COG categories), Genes on reverse strand (color by COG categories), RNA genes (tRNAs green, rRNAs red, other RNAs black), GC content, GC skew.

**Figure 6:**
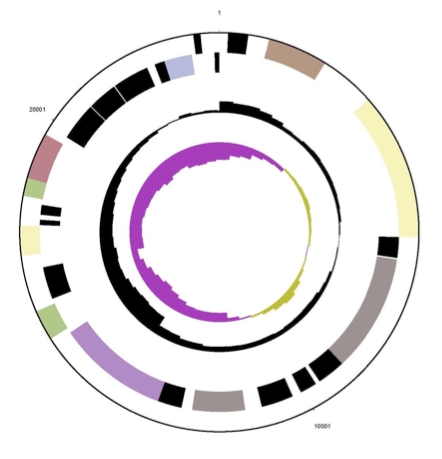
Graphical circular map of second plasmid of strain Spyr1. From outside to the center: Genes on forward strand (color by COG categories), Genes on reverse strand (color by COG categories), RNA genes (tRNAs green, rRNAs red, other RNAs black), GC content, GC skew.

The genome consists of a 5,547,747 bp long circular chromosome with a G+C content of 68% and two plasmids (Figure 4, Figure 5, Figure 6, and Table 3). The larger is 211,864 bp long with 66% G+C content and the smaller 23,681 bp with 64% G+C content.  Of the 5,434 genes predicted, 5,379 were protein-coding genes, and 55 RNAs; 30 pseudogenes were also identified. The majority of the protein-coding genes (67.3%) were assigned a putative function while the remaining ones were annotated as hypothetical proteins. The distribution of genes into COGs functional categories is presented in Table 4.

**Table 3:** Genome Statistics

**Attribute**	**Value**	**% of Total**
Genome size (bp)	5,783,292	100.00%
DNA coding region (bp)	5,256,086	90.88%
DNA G+C content (bp)	3,918,840	67.76%
Number of replicons	1	
Extrachromosomal elements	2	
Total genes	5,434	100.00%
RNA genes	55	1.01%
rRNA operons	2	
Protein-coding genes	5,379	98.99%
Pseudo genes	30	0.55%
Genes with function prediction	3,657	67.30%
Genes in paralog clusters	403	7.42%
Genes assigned to COGs	4,038	74.31%
Genes assigned Pfam domains	4,188	77.07%
Genes with signal peptides	1,617	29.76%
Genes with transmembrane helices	1,185	33.80%
CRISPR repeats	0	

**Table 4:** Number of genes associated with the general COG functional categories

**Code**	**value**	**%age**	**Description**
J	154	3.4	Translation, ribosomal structure and biogenesis
A	20	0.4	RNA processing and modification
K	398	8.7	Transcription
L	305	6.7	Replication, recombination and repair
B	1	0.0	Chromatin structure and dynamics
D	34	0.7	Cell cycle control, cell division, chromosome partitioning
Y	0	0.0	Nuclear structure
V	46	1.0	Defense mechanisms
T	193	4.2	Signal transduction mechanisms
M	176	3.9	Cell wall/membrane/envelope biogenesis
N	10	0.2	Cell motility
Z	1	0.0	Cytoskeleton
W	0	0.0	Extracellular structures
U	38	0.8	Intracellular trafficking, secretion and vesicular transport
O	132	2.9	Posttranslational modification, protein turnover, chaperones
C	303	6.6	Energy production and conversion
G	198	4.3	Carbohydrate transport and metabolism
E	320	7.0	Amino acid transport and metabolism
F	81	1.8	Nucleotide transport and metabolism
H	170	3.7	Coenzyme transport and metabolism
I	412	9.0	Lipid transport and metabolism
P	216	4.7	Inorganic ion transport and metabolism
Q	362	7.9	Secondary metabolites biosynthesis, transport and catabolism
R	636	14.0	General function prediction only
S	351	7.7	Function unknown
-	1,396	25.7	Not in COGs
